# Drought Modulates Root–Microbe Interactions and Functional Gene Expression in Plateau Wetland Herbaceous Plants

**DOI:** 10.3390/plants14152413

**Published:** 2025-08-04

**Authors:** Yuanyuan Chen, Shishi Feng, Qianmin Liu, Di Kang, Shuzhen Zou

**Affiliations:** 1 Key Laboratory of Southwest China Wildlife Resources Conservation (Ministry of Education), China West Normal University, Nanchong 637009, China; cyuanyuan0708@163.com (Y.C.); fss163@163.com (S.F.); qianminl670@163.com (Q.L.); zousz@foxmail.com (S.Z.); 2 Key Laboratory of Environmental Science and Biodiversity Conservation (Sichuan Province), China West Normal University, Nanchong 637009, China

**Keywords:** functional genes, moisture status, plant root traits, plant–microbe interactions

## Abstract

In plateau wetlands, the interactions of herbaceous roots with ectorhizosphere soil microorganisms represent an important way to realize their ecological functions. Global change-induced aridification of plateau wetlands has altered long-established functional synergistic relationships between plant roots and ectorhizosphere soil microbes, but we still know little about this phenomenon. In this context, nine typical wetlands with three different moisture statuses were selected from the eastern Tibetan Plateau in this study to analyze the relationships among herbaceous plant root traits and microbial communities and functions. The results revealed that drought significantly inhibited the accumulation of root biomass and surface area as well as the development of root volumes and diameters. Similarly, drought significantly reduced the diversity of ectorhizosphere soil microbial communities and the relative abundances of key phyla of archaea and bacteria. Redundancy analysis revealed that plant root traits and ectorhizosphere soil microbes were equally regulated by soil physicochemical properties. Functional genes related to carbohydrate metabolism were significantly associated with functional traits related to plant root elongation and nutrient uptake. Functional genes related to carbon and energy metabolism were significantly associated with traits related to plant root support and storage. Key genes such as CS,gltA, and G6PD,zwf help to improve the drought resistance and barrenness resistance of plant roots. This study helps to elucidate the synergistic mechanism of plant and soil microbial functions in plateau wetlands under drought stress, and provides a basis for evolutionary research and conservation of wetland ecosystems in the context of global change.

## 1. Introduction

Wetlands play an irreplaceable and important role in providing water for human beings, maintaining ecological balance and reducing surface source pollution. However, the ecological functions of wetlands are easily affected by both natural factors and human activities [1]. In the context of global change, an increasing number of studies have shown that drought has become a key factor affecting the ecological functions of wetlands, especially ecologically fragile high-elevation wetlands, where the effects of drought are more significant [2]. Wetland ecological functions are related to wetland organisms, and in high-elevation wetlands, herbaceous plants and soil microorganisms are the main providers of ecological functions, and their dynamic change patterns and interaction mechanisms under drought conditions are hot spots in ecological research [3]. The Tibetan Plateau is a sensitive and early warning region for global climate change [4,5], and its special topographic conditions have nurtured rich and diverse alpine wetland ecosystems [6,7]. However, in recent decades, owing to the combined effects of climate change and human activities, the wetland area of the Tibetan Plateau has shown a gradual decrease in area, and the problem of nitrogen pollution has also increased [8,9]. In this context, in-depth investigations of wetland herbaceous plant–soil interactions are highly valuable and urgently needed.

The root system is a key organ for plant water and nutrient uptake and is able to acutely sense and respond to changes in soil moisture. Under water stress conditions, root structure and function are significantly altered, which in turn affects the nutrient uptake efficiency and ultimately restricts plant growth and development [10]. Plant root functional traits are attributes that reflect key plant life activities such as resource acquisition, water transport and nutrient cycling [11]. Plant root–soil microbial interactions are important drivers of ecosystem function and stability [12]. Plant roots influence the structures and functions of soil microbial communities by secreting organic matter to provide soil microorganisms with carbon sources and energy, and by altering soil abiotic properties and microenvironments [13,14,15]. As feedbacks, soil microorganisms provide essential nutrients for plant growth by decomposing and transforming nutrients, and by regulating plant growth and community structures through symbiotic, parasitic or antagonistic relationships [13,16,17,18]. These plant‒soil microbial interactions form “plant‒soil feedbacks” (PSFs) [16,19,20,21,22], which are critical for vegetation growth, community diversity and stability, and ecosystem functioning. PSFs can proved either positive or negative feedback that affects the competitive advantage and diversity of communities by promoting or inhibiting plant growth, respectively [16,19,20,21]. Soil microorganisms, as key players in PSF, are key drivers of plant diversity and productivity by altering soil physicochemical properties and directly or indirectly affecting plant growth [17,20,21]. Currently, many studies have focused on the interactions among plant roots and soil microorganisms, but most have concentrated on a single ecosystem type or short-term experimental conditions; however, for ecologically fragile ecosystems such as plateau wetlands, which are significantly affected by climate change, especially under prolonged drought stress, the mechanisms of the interactions among plant roots and soil microorganisms and their impacts on ecosystem functions are still unclear. In addition, there is a lack of in-depth studies on the mechanisms of microbial functional genes in plant root resistance. In particular, the ectorhizosphere soils, which are a key area for interactions among plant roots and soil microorganisms, significantly affect plant access to nutrients, improve soil structures, and maintain plant health through the synergistic actions of root secretions and microorganisms, and are of irreplaceable importance for sustainable agriculture and ecosystem stability [23].

To elucidate the dynamics of and interactions among plant root functions and ectorhizosphere soil microbial compositions and functions under the influence of drought, the present study was conducted to determine the characteristics of the plant root traits and ectorhizosphere soil microbial communities of herbaceous plants in nine wetland sample plots located on the Western Sichuan Plateau, and to explore the changes in microbial function and their relationships with plant root traits through macrogenome technology.

We hypothesized that (1) drought significantly inhibits herbaceous root biomass accumulation, root surface area, and volume development, and reduces the diversities of ectorhizosphere soil microbial communities and the relative abundance of key phyla; (2) soil physicochemical properties such as soil organic matter (SOM), total nitrogen (TN), total phosphorus (TP), and pH play key roles in regulating herbaceous plant root traits and ectorhizosphere soil microbial community structure, and changes in these factors further affect the interactions between plant roots and ectorhizosphere soil microbes; and (3) there is a significant correlation between plant root traits of herbaceous plants and the structural and functional genes of ectorhizosphere soil microbial communities, and specific microbial functional genes are able to influence plant adaptations to drought stress by regulating the growth and nutrient uptake capacity of plant roots. The findings of this study can provide a theoretical basis for elucidating the formation of a new pattern of wetland biotic interactions in the context of climate change, as well as support wetland conservation and restoration.

## 2. Results

### 2.1. Effects of Different Moisture Statuses on the Root Traits of Herbaceous Plants

#### 2.1.1. Differential Analysis of the Plant Root Traits of Herbaceous Plants at the Community Scale

**Figure 1:**
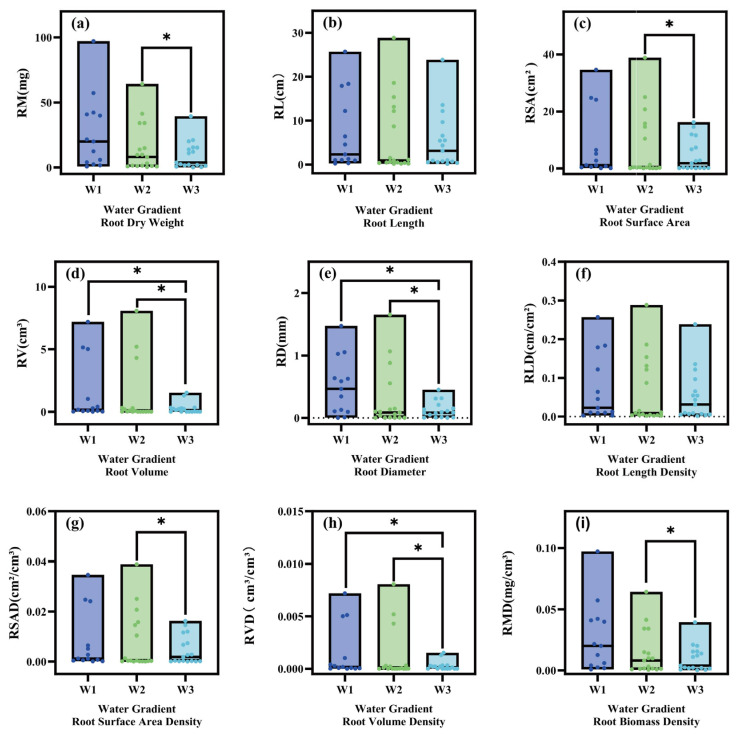
Variability in plant root traits of herbaceous plants in different communities. W1: water-rich wetland, W2: water-scarce wetland, W3: aridized wetland; subfigure (**a**) shows the effect of moisture status on root biomass (RM, mg); subfigure (**b**) shows the effect of moisture status on root length (RL, cm); subfigure (**c**) shows the effect of moisture status on root surface area (RSA, cm^2^); subfigure (**d**) shows the effect of moisture status on root volume (RV, cm^3^); subfigure (**e**) shows the effect of moisture status on root diameter (RD, mm); subfigure (**f**) shows the effect of moisture status on root length density (RLD, cm/cm^3^); subfigure (**g**) shows the effect of moisture status on root surface area density (RSAD, cm^2^/cm^3^); subfigure (**h**) shows the effect of moisture status on root volume density (RVD, cm^3^/cm^3^); subfigure (**i**) shows the effect of moisture status on root biomass density (RMD, mg/cm^3^); (*: *p* < 0.05).

Moisture status had a significant effect on plant root traits, with several indicators of the root system showing clear trends under different moisture statuses (e.g., W1, W2, and W3) (Figure 1). RM and RMD were highest in W1 and significantly lower in W2 and W3, indicating that water conditions have an important effect on root biomass accumulation (Figure 1a,f). RL and RLD did not vary much among the three moisture statuses, indicating a relatively small response of RL to moisture status (Figure 1b,f). The RSA and RSAD did not change significantly under W1 and W2 and were significantly lower than under W3, with significant differences between W2 and W3 (Figure 1c,g), and no significant differences between W1 and W3, suggesting that relatively high moisture conditions favored the expansion of root surface areas, whereas aridification of water-deficient wetlands had a significant inhibitory effect on the root surface areas. RV, RD and RVD showed similar trends among the three moisture statuses, with no significant changes in W1 and W2 and significant decreases in W3 (Figure 1d,e,h), suggesting that moisture conditions have an important effect on root volume versus root diameter development.

#### 2.1.2. Synergies and Trade-Offs in Herbaceous Plant Root Traits

**Figure 2:**
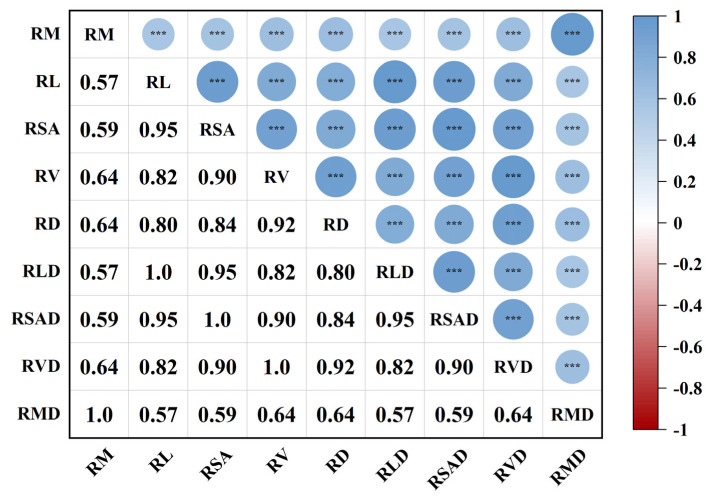
Harmonized trade-offs for plant root traits (***: *p* < 0.001).

Figure 2 shows the correlations among different root trait indicators of herbaceous plants. The results revealed that most of the plant root traits were positively correlated (absolute value of correlation coefficient ≥ 0.8 and *p* ≤ 0.001), such as the correlation coefficients of RL and RLD, RSA and RSAD, and RV and RVD, which were 1.0. RL was also highly positively correlated with RSA, RV, and RD, which indicated that the trends of the changes in the root traits of the plants were highly consistent. There were some moderate positive correlations (absolute values of correlation coefficients between 0.5 and 0.8), such as RM with RL and RSA. Low and weak correlations (absolute values of correlation coefficient < 0.5) were not evident in the figure. Overall, there were strong positive correlations among the plant root traits, which tended to vary synergistically.

#### 2.1.3. Factors Influencing the Plant Root Traits in Herbaceous Plants

**Figure 3:**
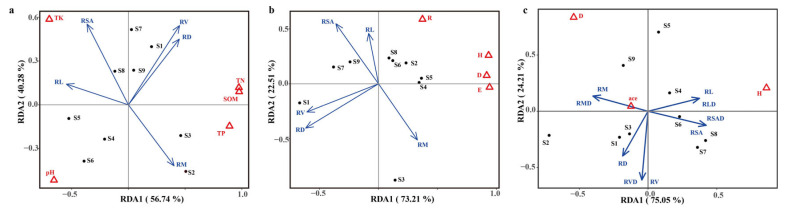
RDA plot of factors influencing plant root traits. S1–S9: numbering of nine sample plots with different moisture status, where S1–S3 are water-rich wetlands (W1), S7–S9 are water-scarce wetlands (W2), and S4–S6 are aridized wetlands (W3). (**a**) RDA plot showing the influence of soil physicochemical properties on plant root trait; (**b**) RDA plot depicting the influence of plant diversity indices on plant root traits; (**c**) RDA plot showing the influence of soil microbial diversity indices on plant root traits. RM: root biomass, RL: root length, RSA: root surface area, RV: root volume, RD: root diameter, SOM: soil organic matter; TN: total nitrogen; TP: total phosphorus; TK: total potassium; pH: soil pH; D: Simpson diversity index; H: Shannon‒Wiener diversity index; E: Pielou evenness index; R: species richness index. Arrows indicate the correlation between different influencing factors and plant root traits; longer arrow indicates longer correlation, and the direction of the arrow indicates positive correlation.

The interrelationships among ectorhizosphere soil physicochemical factors, herbaceous plant diversity indices, ectorhizosphere soil microbial diversity indices, and plant root traits were further analyzed via redundancy analysis (Figure 3). A total of 97.02% of the variance was explained by the two ordinations in the redundancy analysis of ectorhizosphere soil physicochemical factors and plant root traits (Figure 3a). RV was significantly correlated with pH (r2 = 0.7133, *p* = 0.004); TN (r2 = 0.6174, *p* = 0.012); and SOM (r2 = 0.5783, *p* = 0.017). RD was significantly correlated with TN (r2 = 0.7037, *p* = 0.005); SOM (r2 = 0.6743, *p* = 0.007); TP (r2 = 0.5830, *p* = 0.018); and pH (r2 = 0.5441, *p* = 0.023). In addition, RM was significantly correlated with TK (r2 = 0.6176, *p* = 0.012); TN (r2 = 0.5858, *p* = 0.016); SOM (r2 = 0.5834, *p* = 0.017); and TP (r2 = 0.5830, *p* = 0.017). RSA, RV, and RD are negatively correlated with pH, and RM was also negatively correlated with TK. pH, SOM, TN, TP, and TK were the key ectorhizosphere soil physicochemical factors that affect plant root traits. In the redundancy analysis of the diversity indices of herbaceous plants with respect to plant root traits, the two ordination axes explained 95.72% of the variance (Figure 3b). The RSA was significantly correlated with D (r2 = 0.6993, *p* = 0.005); H (r2 = 0.5937, *p* = 0.015); and E (r2 = 0.5926, *p* = 0.015). RV was significantly correlated with H (r2 = 0.6738, *p* = 0.007); D (r2 = 0.6253, *p* = 0.011); and E (r2 = 0.5054, *p* = 0.032). RD was significantly correlated with H (r2 = 0.5568, *p* = 0.021). RSA, RV, and RD were all negatively correlated with biotic factors, and the key herb diversity indices affecting plant root traits were D, H, and E. In the redundancy analysis of ectorhizosphere soil microbial diversity indices with plant root traits, the two ordination axes explained 99.26% of the variance (Figure 3c). RM and RMD were significantly correlated with H (r2 = 0.6690, *p* = 0.007). RM, RMD, and H were negatively correlated, and the key ectorhizosphere soil microbial index affecting plant root traits was H. The other ectorhizosphere soil microbial diversity indices had no significant effect on plant root traits.

### 2.2. Effects of Different Moisture Statuses on Ectorhizosphere Soil Microbial Communities

#### 2.2.1. Analysis of Ectorhizosphere Soil Microbial Community Diversities

The alpha diversities of the soil archaeal, bacterial, and fungal communities were analyzed under different moisture conditions (Table 1). As shown in Table 1, different moisture statuses had different degrees of influence on the alpha diversities of the soil archaeal, bacterial, and fungal communities. Overall, there were no significant differences in the Chao1 index, Shannon‒Wiener index, or Simpson index among the archaeal, bacterial, and fungal communities under different moisture statuses.

**Figure 4:**
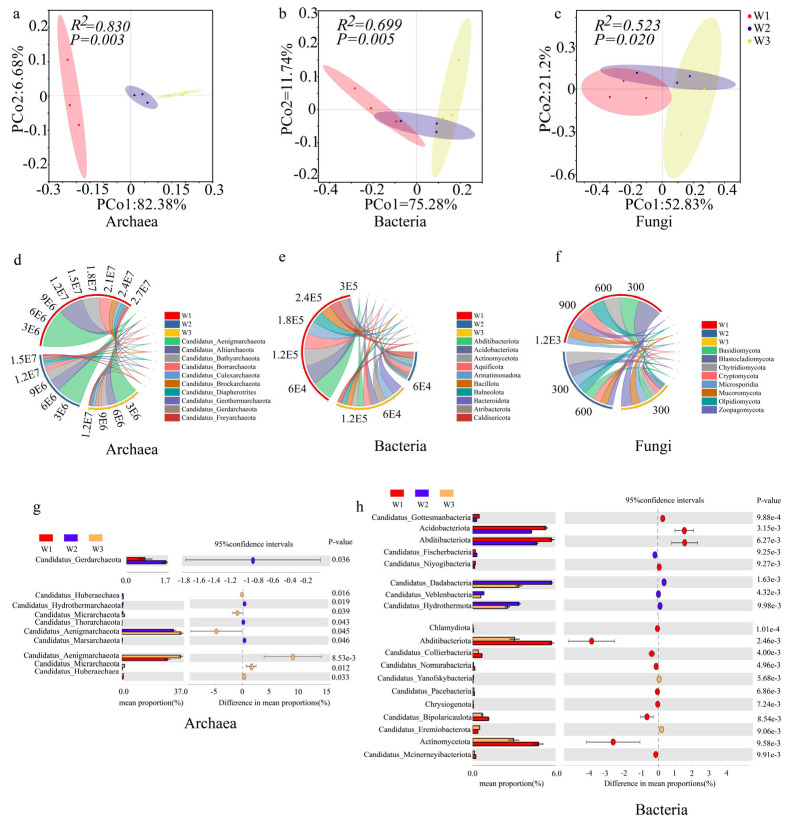
PCoA-based soil microbial community structure and its abundance pattern at key phylum level. PCoA: Principal Coordinate Analysis, used to show the similarity of soil microbial community structure in different sites; different colors and shapes of dots represent the sample sites under different moisture status: red is W1, purple is W2, yellow is W3, and the further distance between the dots indicates the greater differences in the microbial community structure; (**a**) is the PCoA diagram for archaea; (**b**) is the PCoA diagram for bacteria; (**c**) is the PCoA diagram for fungi. Chordal plots show the relative abundance of the major phyla of archaea (**d**), bacteria (**e**), and fungi (**f**) under different moisture status: red is W1, blue is W2, yellow is W3;STAMP analysis identifies key taxa and changes in relative abundance of key phyla at the phylum level for archaea (**g**) and bacteria (**h**): red is W1, blue is W2, yellow is W3.

PCoA was performed for the soil archaeal, bacterial, and fungal communities under the different treatments based on Bray‒Curtis distances (Figure 4). In the PCoA analysis of the archaeal communities, the cumulative explanation of the variance of the two principal components was 89.06%, with a significant difference between the groups (*R*^2^ = 0.830, *p* = 0.003; Figure 4a), and the archaeal communities were segregated into W1, W2, and W3 moisture statuses. In the PcoA of the bacterial communities, the cumulative variance that was explained by the two principal components was 87.02%, with a significant difference between groups (*R*^2^ = 0.699, *p* = 0.005; Figure 4b); the bacterial communities under the three moisture statuses had high degrees of aggregation, with high degrees of overlap between different communities. In the PcoA of fungal communities, the cumulative variance that was explained by the two principal components was 74.03%, and the difference between groups was significant (*R*^2^ = 0.532, *p* = 0.020; Figure 4c), with fungal communities clustered together under the three moisture statuses and high degrees of overlap between the different communities.

#### 2.2.2. Analysis of Ectorhizosphere Soil Microbial Community Structure and Its Key Phyla

Figure 4 shows the relative abundances of ectorhizosphere soil microorganisms at the level of major and key phyla; and the distribution characteristics of archaea (Figure 4d,g), bacteria (Figure 4e,h), and fungi (Figure 4f) in W1, W2, and W3, respectively, were analyzed. The chord plots (Figure 4d–f) show that archaea and fungi in W1 contain a variety of phyla and large relative abundances, with an overall trend of decreasing microbial abundance with decreasing moisture. In contrast, bacteria had the lowest abundances in the W2 moisture condition, which may be because bacteria need more time to acclimatize under drought conditions. The gates of the three microorganisms were subsequently analyzed (Figure 4g,h). The results revealed that Candidatus_Gerdarchaeota differed significantly under the W1 and W2 moisture statuses of archaea; under the W2 and W3 moisture statuses, Candidatus_Huberaechaea, Candidatus_Hydrothermarchaeota, Candidatus_Micrarchaeota, Candidatus_Thorarchaeota, Candidatus_Aenigmarchaeota, and Candidatus_Marsarchaeota differed significantly; under the W1 and W3 moisture statuses, Candidatus_Aenigmarchaeota, Candidatus_Micrarchaeota, and Candidatus_Huberaechaea differed significantly; for the bacterial phyla, Candidatus_Gottesmanbacteria, Acidobacteriota, Abditibacteriota, Candidatus_Fischerbacteria, and Candidatus_Niyogi bacteria differed significantly under the W1 and W2 moisture statuses; and Candidatus_Dadabacteria, Candidatus_Veblenbacteria, and Candidatus_Hydrothermota differed significantly under W2 and W3 moisture status; Chlamydiota, Abditibacteriota, Candidatus_Collierbacteria, Candidatus_Nomurabacteria, Candidatus_Yanofskybacteria, Candidatus_Pacebacteria, Chrysiogenota, Candidatus_Bipolaricaulota, Candidatus_Eremacteria, Actinomycetota, and Candidatus_Mcinerneyibacteriota were significantly different under W1 and W3 moisture status; the fungal phyla did not differ significantly across moisture status. In this study, the archaeal phyla Candidatus_Aenigmarchaeota and Candidatus_Marsarchaeota showed significant decreases under drought stress, while Candidatus_Borrarchaeota and Candidatus_Huberarchaea had higher relative abundances in water-rich wetlands.

#### 2.2.3. Relationships Among Ectorhizosphere Soil Microbial Community Compositions and Environmental Factors

**Figure 5:**
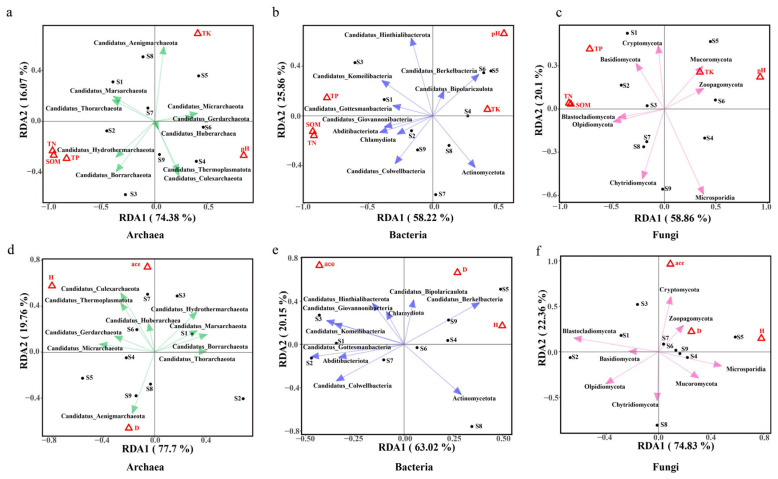
Relationship between key phyla of ectorhizosphere soil microorganisms and environmental factors. The figure shows the correlation between key phyla of soil microorganisms and environmental factors through redundancy analysis (RDA). S1–S9: numbering of nine sample plots with different moisture status, where S1–S3 are water-rich wetlands (W1), S7–S9 are water-scarce wetlands (W2), and S4–S6 are aridized wetlands (W3). (**a**,**d**) are RDA plots showing the relationships between archaeal key phyla and soil physicochemical properties and soil microbial diversity indices, represented in green; (**b**,**e**) are RDA plots showing the relationships between bacterial key phyla and soil physicochemical properties and soil microbial diversity indices, represented in blue; (**c**,**f**) are RDA plots showing the relationships between fungal key phyla and soil physicochemical properties and soil microbial diversity indices, represented in pink; Different colored arrows indicate the correlation between environmental factors and microbial phyla, with longer arrows indicating stronger correlations, and arrow direction indicating positive or negative correlations. SOM: soil organic matter; TN: total nitrogen; TP: total phosphorus; TK: total potassium; pH: soil pH; D: Simpson’s diversity index, which is used to measure the diversity of the microbial community. ace: Ace index, used to estimate the species richness of the microbial community. H: Shannon‒Wiener diversity index, used to measure the diversity of the microbial community.

Figure 5 redundancy analysis revealed that for the archaeal factors at the phylum level (Figure 5a,d), SOM was positively correlated with Candidatus_Borrarchaeota, Candidatus_Hydrothermarchaeota, Candidatus_Huberarchaea, Candidatus_Thorarchaeota, Candidatus_Aenigmarchaeota, and Candidatus_Marsarchaeota were highly significantly positively correlated, and Candidatus_Micrarchaeota and Candidatus_Thermoplasmatota were significantly negatively correlated; TP was significantly positively correlated with Candidatus_Huberarchaea, Candidatus_Borrarchaeota, Candidatus_Aenigmarchaeota, Candidatus_Thorarchaeota, Candidatus_Hydrothermarchaeota, Candidatus_Marsarchaeota, Candidatus_Thermoplasmatota, and Candidatus_Culexarchaeota; TN was significantly positively correlated with Candidatus_Borrarchaeota, Candidatus_Thorarchaeota, Candidatus_Hydrothermarchaeota, Candidatus_Huberarchaea, Candidatus_Aenigmarchaeota, Candidatus_Marsarchaeota, and Candidatus_Micrarchaeota were was significantly positively correlated; pH was significantly positively correlated with Candidatus_Micrarchaeota and Candidatus_Gerdarchaeota; TK was significantly negatively correlated with Candidatus_Borrarchaeota; and D was significantly positively correlated with Candidatus_Thorarchaeota and Candidatus_Marsarchaeota. For bacteria at the phylum level (Figure 5b,e), TP was associated with Candidatus_Gottesmanbacteria, Candidatus_Berkelbacteria, Candidatus_Giovannonibacteria, Actinomycetota, Chlamydiota, and Abditibacteriota, which were highly significantly positively correlated with Candidatus_Colwellbacteria, Candidatus_Komeilibacteria, Candidatus_Bipolaricaulota, and Candidatus_Hinthialibacterota. SOM and TN were highly significantly positively correlated with all key bacterial phyla; TK was significantly positively correlated with Candidatus_Colwellbacteria and Actinomycetota; D was significantly positively correlated with Candidatus_Bipolaricaulota and Candidatus_Berkelbacteria; and H was significantly positively correlated with Candidatus_Hintbialibacterota and Candidatus_Giovannonibacteria. For fungi at the phylum level (Figure 5c,f), TP was significantly and positively correlated with Cryptomycota, Basidiomycota, and Mucoromycota; SOM was significantly negatively correlated with Zoopagomycota and Olpidiomycota; pH was significantly negatively correlated with Chytridiomycota; TK was significantly positively correlated with Cryptomycota and Basidiomycota; D was significantly positively correlated with Cryptomycota and Basidiomycota; Ace was significantly positively correlated with Zoopagomycota and Olpidiomycota; and H was significantly positively correlated with Zoopagomycota and Olpidiomycota.

### 2.3. Interactions Among Herbaceous Plant Root Traits and Microbial Communities

#### 2.3.1. Correlations of Plant Root Traits with the Relative Abundances of Species at the Microbial Keystone Phylum Level

**Figure 6:**
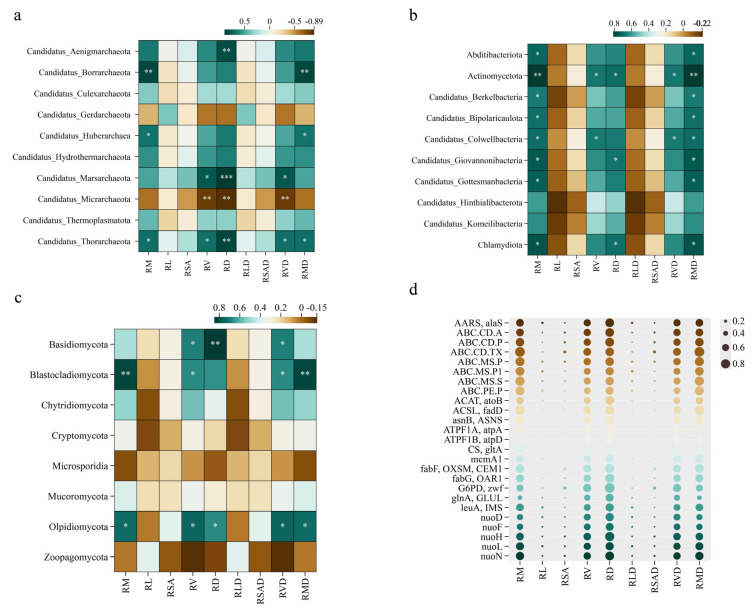
Heat map of correlation between plant root traits and relative abundance of species at the key phylum level and bubble map of key functional genes of significantly related phyla. (**a**) Heat map displaying the correlation between plant root traits and the relative abundance of species at the key phylum level for Archaea; (**b**) Heat map showing the correlation between plant root traits and the relative abundance of species at the key phylum level for Bacteria; (**c**) Heat map illustrating the correlation between plant root traits and the relative abundance of species at the key phylum level for Fungi; (**d**) Bubble map representing the key functional genes of significantly related phyla. Bubbles vary in size from smallest to largest, representing correlations ranging from 0.2 to 0.8. The heat maps in (**a**–**c**) use a color scale where blue indicates positive correlations and yellow indicates negative correlations. The bubble map in subfigure d provides a visual representation of the strength and significance of correlations between plant root traits and key functional genes. (*: *p* < 0.05, **: *p* < 0.01, ***: *p* < 0.001).

As shown in Figure 6, there were correlations between different microbial phyla and plant root traits. At the level of archaea (Figure 6a), Candidatus_Aenigmarchaeota was significantly positively correlated with RD; both Candidatus_Borrarchaeota and Candidatus_Huberarchaea were significantly positively correlated with RM and RMD; Candidatus_Marsarchaeota was significantly positively correlated with RV, RD, and RVD; Candidatus_Thorarchaeota was significantly positively correlated with RM, RV, RD, RVD, and RMD; conversely, Candidatus_Micrarchaeota was significantly negatively correlated with RV, RD, and RVD. At the bacterial phylum level (Figure 6b), Abditibacteriota, Candidatus_Berkelbacteria, Candidatus_Bipolaricaulota, and Candidatus_Gottesmanbacteria were significantly positively correlated with RM and RMD; Candidatus_Giovannonibacteria and Chlamydiota were significantly positively correlated with RM, RD, and RMD; Actinomycetota was significantly positively correlated with RM, RV, RD, RVD, and RMD; and Candidatus_Colwellbacteria was significantly positively correlated with RM, RV, RVD, and RMD. At the fungal phylum level (Figure 6c), Basidiomycota was significantly positively correlated with RV, RD, and RVD; Blastocladiomycota was significantly positively correlated with RM, RD, RVD, and RMD; and Olpidiomycota was significantly positively correlated with RM, RV, RD, RVD, and RMD.

#### 2.3.2. Relationships Among Plant Root Traits and Functional Microbial Genes

The role of genes in root development and function was explored by analyzing the correlations among different genes and plant root traits (Figure 6d) and in relation to the function of genes in metabolic pathways (Table S1 in the Supplementary Materials). The results revealed that the correlations of genes with plant root traits were closely related to their functions in metabolic pathways. The fabG,OAR1, G6PD,zwf, leuA,IMS, fabF,OXSM,CEM1 genes were highly correlated with the RL and RSA traits; the CS,gltA gene was highly correlated with RM, RL, RSA, and RV traits; the nuoD and nuoF genes were highly correlated with the RMD trait; and the glnA,GLUL gene was highly correlated with the RL and RSA traits. These findings underscore the importance of specific microbial functional genes in shaping plant root traits under different moisture statuses, which is crucial for elucidating plant drought resilience mechanisms.

**Figure 7:**
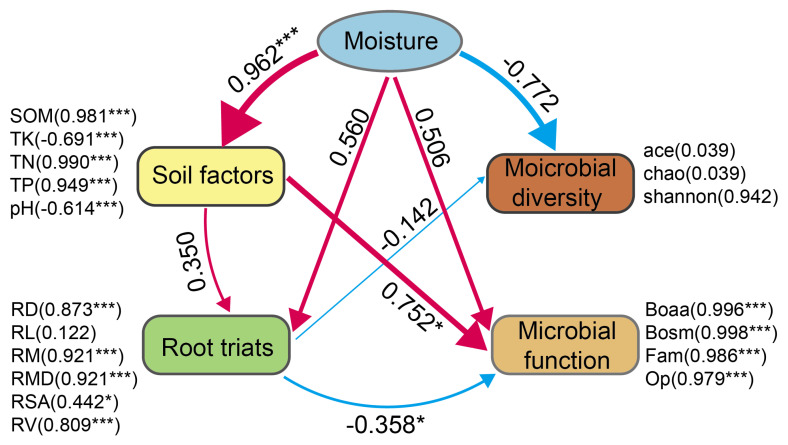
Structural equation modeling of microbial diversity and functioning. The model revealed indirect effects of moisture status on herbaceous plant root traits and soil microbial functions through soil factors such as soil organic matter—SOM, total nitrogen—TN, total phosphorus—TP, and pH, as well as direct and indirect causal relationships between these factors (model goodness-of-fit—GOF = 0.77). One-way arrows indicate the proposed causal relationships, and path coefficients indicate the strength of these relationships; positive correlations are indicated by red arrows and negative correlations by blue-colored arrows, with the width of the arrow corresponding to the strength of the relationship. In the model, SOM, TN, TP, TK, and pH were the key soil factors affecting plant root traits and microbial function; plant root traits had a significant positive effect on microbial function, and moisture status also directly affected microbial function significantly and positively. (*: *p* < 0.05, ***: *p* < 0.001.)

Through structural equation modeling (Figure 7), the moisture status significantly affected soil factors, including SOM, TN, TP, and pH, with path coefficients of 0.962, 0.560, and 0.506 for SOM, TN, and TP, respectively, which were all significant at the *p* < 0.001 level, and path coefficients of −0.614 for pH, which were also significant at the *p* < 0.001 level. These soil factors significantly affected the root characteristics, with a path coefficient of 0.350 and a significance level of *p* < 0.001. The root characteristics had a significant positive effect on microbial functions, with a path coefficient of 0.752, which was significant at the *p* < 0.05 level. In addition, the moisture status also had a significant positive effect directly on microbial functions, with a path coefficient of 0.560, which was significant at the *p* < 0.001 level.

## 3. Discussion

### 3.1. Response of Plant Root Traits to Moisture Status

The root system is an important organ for the exchange of material and energy between the plant body and the external environment and is in direct contact with the soil, playing a key role in anchoring and supporting the plant body, as well as absorbing mineral nutrients and water from the soil [24,25]. The responses of plant root traits to moisture status are important for plant growth. The moisture status had a significant effect on the plant root traits of the herbaceous plants (Figure 1), especially the RM and RMD, which reached their highest values in W1 and decreased significantly in W3. This suggests that plants tend to accumulate more biomass in well-watered environments, whereas biomass accumulations in root systems are inhibited under drought conditions. In addition, RSA and RV significantly declined in W3, indicating the inhibitory effect of arid conditions on the root expansion capacity. This change may be related to the fact that plants prioritize the allocation of resources to other survival strategies (e.g., water storage or stress tolerance) under drought conditions. In contrast, RD and RLD showed lower variations across moisture statuses, suggesting that the responses of these traits to moisture changes were relatively stable, possibly reflecting the conservative strategy of plants under different moisture statuses. Other studies have reached conclusions similar to ours. A study investigating the drought stress of megagrass revealed that with increasing stress, the RSA and RV of megagrass subsequently decreased, the RM and root vigor weakened, especially under severe drought stress, and the degree of decrease of these indices became more significant [26]. Another study also revealed that under certain stresses, plants allocate more biomass to the root system, and the belowground biomass increases with the degree of stress; however, under severe drought stress, the root mass decreases drastically [27]. Moreover, megagrass preferentially allocates more assimilates to the root system when faced with water deficit, thereby increasing the root‒crown ratio and increasing the surface area of the root system to increase water uptake [10].

The negative correlation between Shannon‒Wiener diversity and root biomass (RM, RMD) may be attributed to several factors, including altered carbon allocation under microbial stress and changes in the soil microenvironment due to high root density [28]. High root biomass environments may lead to increased competition for resources among microbes, potentially reducing microbial diversity [29]. Additionally, dense root systems can alter soil porosity and oxygen availability, creating conditions that may favor certain microbial taxa over others [30,31].

In water-rich environments, plants efficiently absorb nutrients by expanding the surface areas and volumes of their root systems to support their growth and productivity [32]. In contrast, in arid environments, root contraction may be a strategy for plants to reduce resource consumption to prioritize the allocation of resources to other survival functions (e.g., water storage or stress tolerance) [33]. We found that SOM, TN, TP, and pH were the key factors affecting plant root traits. RV was significantly and positively correlated with TN and SOM, and RD was significantly and positively correlated with TN, SOM, and TP (Figure 3a). These results are consistent with those of most previous studies [12,34,35], showing that increased SOM and TN may promote root expansion and biomass accumulation by providing more nutrients and improving the soil structure. In addition, plant diversity (e.g., Simpson’s index and Shannon‒Wiener index) was significantly correlated with plant root traits (e.g., RSA and RV). These findings suggest that plant community diversity may influence soil resource utilization efficiency by regulating plant root traits. Related studies also support the idea that diverse plant communities may utilize soil resources more efficiently through different combinations of plant root traits, thereby increasing ecosystem productivity and stability [36]. Thus, soil factors and plant diversity together influence plant root traits, which in turn affect the plant utilization efficiency of soil resources and the overall functioning of the ecosystem.

### 3.2. Ecological Functions of Soil Microbial Communities

Soil microorganisms are the most active factors in soil ecosystems; play important roles in soil formation and development, decomposition of soil organic matter, material and energy inputs, nutrient transformation, and fertility evolution; and are important links in the ecological chain of the material cycle, which mainly includes bacteria, fungi, and archaea [37]. This study revealed that the composition and structures of soil fungal communities changed significantly with changes in soil moisture conditions (Figure 4), with the highest abundances of key phyla of archaea, bacteria, and fungi occurring in W1, and a significant decrease in the abundances of key phyla of archaea, bacteria, and fungi occurring in W3, which may be related to the sensitivity of fungi to drought conditions [38]. In contrast, bacteria and archaea were more adaptable to different moisture conditions, but still presented some changes in community structure. In one study, the sizes and structures of the soil archaeal communities in the plateau wetlands of the Tibetan Plateau in dry and wet years were investigated via real-time quantitative PCR and TRFLP techniques, and the results revealed that the number of soil archaeal communities decreased significantly in dry years [39], which is similar to the results of the present study. However, another study using a plate counting technique to implement water stress experiments on round-leaved Cassia potted plants reported that water stress significantly increased the numbers of bacteria, fungi, actinomycetes, and nitrogen-fixing bacteria in the inter-root soil, and that the number of soil fungi and actinomycetes tended to increase with increasing stress duration [40]. The differences in these findings reveal a complex response mechanism of soil microbial communities to water stress, which is influenced by a combination of multiple factors, including ecosystem type, soil texture, and the initial structure of the microbial community.

Although archaea and fungi account for a relatively small proportion in the microbial community, they play a key role in plant root function and drought resistance [41]. The abundance of the Candidatus_Aenigmarchaeota group significantly decreases under drought stress, which may weaken the ammonia oxidation pathway (such as the expression of the amoA gene), thereby inhibiting the soil nitrogen cycle and affecting the drought resistance of plants [42]. The abundance variation of Basidiomycota is also closely related to soil structure and the root expansion capacity of plants. In water-rich wetlands, its high abundance is conducive to lignin degradation and soil aggregate formation, enhancing soil water retention capacity and plant root expansion capacity. However, under drought conditions, the decrease in its abundance may lead to soil structure deterioration, restrict plant root expansion, and weaken the drought resistance of plants [43].

Soil microorganisms significantly affect the soil organic matter content and nutrient cycling by decomposing organic matter in the soil and converting it to inorganic matter [44]. In the process of decomposing complex organic compounds such as cellulose and lignin, microorganisms are able to improve the soil structure and increase porosity, which in turn enhances soil aeration and water permeability [45]. The physicochemical properties of soil, such as pH and water content, directly affect the growth and metabolic activities of microorganisms [46]. In this study, the effects of soil physicochemical properties on microbial community structures were revealed via redundancy analysis (Figure 5), which revealed that soil organic matter (SOM), total nitrogen (TN), and total phosphorus (TP) were the main determinants of microbial community distributions [47,48].

### 3.3. Relationships Among Plant Root Traits and Ectorhizosphere Soil Microbial Compositions

Plant root traits and microbial interactions are important pathways for the realization of wetland ecosystem functions. Studies have shown that root structural and morphological characteristics significantly affect the structures and functions of ectorhizosphere soil microbial communities [49]. Fine roots may contribute to the diversity and activity of inter-root microbial communities by increasing secretion of root exudates and improving nutrient cycling. In addition, the ectorhizosphere soil microbial diversity is critical for plant health, as it increases interference and competition with plant pathogens, thereby preventing pathogen invasion [50,51]. In this study, RM and RMD were significantly negatively correlated with the Shannon‒Wiener diversity index. This may indicate a greater diversity of microbial communities at lower root biomasses, reflecting a complex mechanism of interactions between plant roots and soil microorganisms. This relationship reveals that plants alter the soil microenvironment through root growth and metabolic activity, which in turn affects the structure of the microbial community [52]. For example, grassland systems with high plant diversity are able to attract more diverse microbial taxa through root secretions, thereby maintaining the ectorhizosphere soil microbial diversity and function [53].

Significant correlations were also shown between plant root traits and relative abundances at the microbial gate level. Studies have shown that plants with different root diameters differ in the composition of their inter-root microbial communities [49], and a growing number of studies have demonstrated that smaller root diameters are associated with an increased diversity of their inter-root microbial communities in a wide range of plants, including natural shrubs, acacia, peach, and poplar, as well as in agriculturally important crops such as maize and wheat. However, in the present study, Candidatus_Aenigmarchaeota in the phylum archaea was significantly positively correlated with RD, whereas Candidatus_Micrarchaeota was significantly negatively correlated with RV, RD, and RVD. These findings suggest that the physical properties of fine roots may limit the colonization of certain microorganisms while providing more favorable conditions for other microorganisms to survive [54]. At the bacterial phylum level, the phyla Abditibacteriota, Candidatus_Berkelbacteria, and Actinomycetota were significantly positively correlated with RM and RMD. Actinomycetes are a group of bacteria with the ability to decompose complex organic matter, which may contribute to the accumulation of root biomass by decomposing organic matter in the soil and releasing nutrients that can be absorbed by plants [55]. At the fungal phylum level, Basidiomycota was significantly and positively correlated with RV, RD, and RVD. Basidiomycota is a group of fungi with a wide range of ecological functions, including the ability to decompose complex organic matter such as lignin and cellulose, and their decomposition products, such as humic acids and polysaccharides, and can combine with soil particles to form water-stable agglomerated structures and soil colloidal complexes, thus improving soil aggregation and stability. In addition, substances such as extracellular polysaccharides that are secreted by microorganisms during the decomposition process contribute to the aggregation of soil particles, which further enhances the agglomerate structure of the soil and provides favorable conditions for the expansion and development of the root system [56].

In summary, material accumulation (e.g., root biomass) in the root systems of herbaceous plants in plateau wetlands was significantly correlated with the diversity of ectorhizosphere soil microorganisms and their relative abundances at the phylum level. Changes in plant root traits, especially increases in root biomass and surface area, can significantly influence the structure and function of soil microbial communities, which in turn promotes soil nutrient cycling and plant growth [48]. Moreover, changes in microbial communities, especially in the relative abundances of certain key phyla, can also enhance plant adaptations to environmental stress by regulating soil nutrient availability and the root microenvironment, and by influencing plant root uptake, support, and expansion functions [49]. The degradation of plant root traits and microbial communities during wetland degradation may lead to impaired soil nutrient cycling and restricted plant growth. Therefore, by increasing the soil organic matter content and improving the soil structure, microbial diversity can be improved, which in turn promotes the growth and development of the root system and ultimately improves the productivity and stability of wetland ecosystems [50].

### 3.4. Relationships Among Root Functions and Ectorhizosphere Soil Microbial Functions

The complex relationship between plant roots and soil microbial functions is important for the functional stability of ecosystems [57]. Microorganisms play a key role in the regulatory, supportive, and provisioning functions of soil ecosystems and are the key link between the aboveground and belowground parts of ecosystems [58,59], which can maintain ecosystem multifunctionality by participating in the processes of decomposition of apoplastic matter, mineralization of organic matter, production of primary matter, and transport of matter and energy between aboveground and belowground communities [60]. This study further revealed the relationships between root function and soil microbial function by analyzing the correlations between plant root traits and microbial function genes. The fabG,OAR1, G6PD,zwf genes presented high correlations with RL and RSA, whereas the CS,gltA gene presented high correlations with RM, RL, RSA, and RV (Figure 6d). The functions of these genes in metabolic pathways is closely related to the development of plant root traits (Supplementary Table S1).

Soil microorganisms are able to participate in metabolic pathways through key genes (e.g., CS,gltA, G6PD,zwf, ACAT,atoB, and mcmA1) to synthesize and secrete metabolites, such as organic acids, which in turn regulate the availability of nutrients to the root system [61,62]. Among these genes, the CS,gltA gene promotes the accumulation of root biomass by regulating carbon metabolism and providing the necessary energy and carbon source to the root system [63], whereas the G6PD,zwf gene provides reducing power and nucleic acid precursors to the root system through the pentose phosphate pathway to support its growth and development [64]. In addition, the synergistic effects of genes related to the biosynthesis of secondary metabolites (e.g., fabG,OAR1, G6PD,zwf) with plant root traits suggest that the plant root system may regulate the composition and function of the microbial community through the secretion of secondary metabolites, resulting in a reciprocal relationship [65]. This reciprocal relationship is reflected not only in the physical structure of the root system but also in the metabolic pathways of the microorganisms. Soil microorganisms also influence root growth and development by regulating the synthesis and distribution of hormones in the plant root system. Some microorganisms are able to secrete phytohormones such as growth hormones to promote root elongation and branching, whereas others may secrete hormones such as abscisic acid to inhibit root overgrowth and thus optimize the root architecture in the soil environment [42]. For example, the G6PD,zwf gene synthesizes glucose-6-phosphate dehydrogenase through the pentose phosphate pathway, which provides cells with reducing power and nucleic acid precursors to support root growth and development. Under adverse conditions, such as drought or saline environments, soil microorganisms are able to enhance the resilience of the plant root system by secreting resistance substances or regulating soil water and nutrient status, helping plants to better adapt to environmental stresses [66,67,68]. For example, the CS,gltA gene drives the tricarboxylic acid cycle (TCA cycle) by synthesizing citrate synthase, which provides energy and carbon skeleton precursors to the root system, thus increasing root resilience.

In summary, there were significant correlations between plant root function genes and ectorhizosphere soil microbial function genes. Microbial functional genes involved in carbon metabolism, secondary metabolite synthesis, and energy metabolism are associated with root traits that may support plant growth and stress tolerance. These associations suggest a potentially mutualistic relationship that may support plant survival under adverse conditions such as drought and also provide an important mechanism to guarantee the stability and functional maintenance of wetland ecosystems.

### 3.5. Potential Applications of Microbial Functions in Wetland Ecological Restoration

In this paper, we found that drought stress significantly modulates the interactions between plant roots and ectorhizosphere soil microbial communities, as well as the expression of functional genes in plateau wetlands. These findings provide an important theoretical basis for ecological restoration strategies of wetlands, especially in plant selection and microbial inoculation. Moisture status was found to have a significant effect on plant root traits, with water-rich wetlands (W1) favoring root biomass accumulation and surface area expansion, while aridized wetlands (W3) significantly inhibited the development of these traits (Figure 1). In addition, plant root traits were closely related to the diversity of ectorhizosphere soil microbial communities (Figure 6), suggesting that selecting plant species with strong root expansion and drought tolerance may be more effective during ecological restoration [69,70]. Plants with higher root surface area and biomass are able to absorb water and nutrients more efficiently under drought conditions, thereby enhancing plant survival and ecosystem stability [42,71].

In this study, we also found that the diversity of ectorhizosphere soil microbial communities and the expression of functional genes differed significantly under different moisture conditions. Particularly, functional genes related to carbon and energy metabolism (e.g., CS,gltA, and G6PD,zwf) were closely related to plant root traits (Figure 6), contributing to drought tolerance and nutrient uptake in plants. These results suggest that enhancing ectorhizosphere soil microbial communities through microbial inoculation may be an effective strategy for wetland restoration. Some studies have found that inoculation with specific microbial strains can significantly improve plant resistance and nutrient uptake [69,72]. In wetland restoration, the introduction of microorganisms with specific functional genes, such as those capable of synthesizing and secreting organic acids (via the CS,gltA gene), may be considered to enhance soil nutrient availability [73,74].

In addition, microbial functional genes identified in this study, such as CS,gltA, and G6PD,zwf, play important roles in plant drought tolerance and nutrient uptake (Figure 6). These genes can be applied to wetland restoration through bioaugmentation techniques. Bioaugmentation is the process of enhancing ecosystem functioning by introducing specific strains or communities of microorganisms into the ecosystem [75]. Some studies have found that enhancing the stress resistance of wetland ecosystems can be achieved by screening and introducing microbial strains with specific functional genes, such as microorganisms capable of synthesizing and secreting stress-resistant substances [76,77]. Meanwhile, improving soil structure and nutrient cycling through microbial inoculation can further promote plant growth and enhance wetland ecosystem restoration [78,79].

In summary, the findings of this study provide an important theoretical basis for strategies related to wetland ecological restoration. The resilience and function of wetland ecosystems can be significantly enhanced by selecting plant species with strong root expansion and drought tolerance, and by introducing microbial strains with specific functional genes. Future studies can further explore the specific application mechanisms of these microbial functional genes in wetland restoration to develop more effective ecological restoration strategies.

## 4. Materials and Methods

### 4.1. Study Area

**Figure 8:**
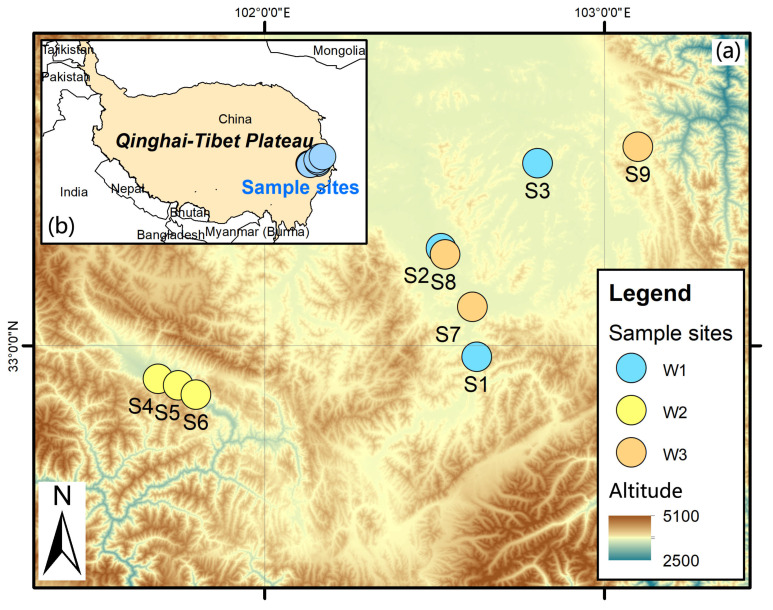
Locations of sample sites. After investigation, we selected nine wetland sample sites with three different moisture statuses: water-rich wetlands (W1), water-scarce wetlands (W2), and aridized wetlands (W3) in the eastern Tibetan Plateau. (**a**) shows the positions of the sample sites we selected on a small scale; (**b**) shows the locations of our sampling points on this large scale in the eastern part of the eastern Tibetan Plateau. For soil sampling, we set up a 20 m × 20 m sample square within each sample plot and randomly selected three sampling points in each sample square. Before collecting soil samples, visible plant and animal debris and apomictic material were removed, and then periapical soil was collected from 10 cm × 10 cm clods using a clean shovel and gently swept near the plant roots into a plastic bag. Sampling was avoided in deep water; if shallow water was encountered, it was drained before sampling. Subsequently, three soil samples from each sample plot were thoroughly mixed to make a composite soil sample from which fine roots and larger plant fragments were removed. Finally, the well-mixed soil samples were divided into two portions: one was naturally air-dried for determination of soil nutrient content, and the other was quickly brought back to the laboratory and stored in a −80 °C refrigerator for subsequent microbial sequencing analysis.

The study area is located on the eastern edge of the Tibetan Plateau (32.73°~32.39° N, 102.34°~102.41° E) (Figure 8). The region has a continental plateau cold–temperate monsoon climate [80], which is characterized by short springs, long winters, and lack of summers, a particularly limited growing season (July to September), and clear boundaries between wet and dry seasons. The average altitude of this place reaches 3507 m, and the average annual temperature is maintained at 1.4 °C, while the annual precipitation reaches 860.8 mm. The vegetation type is typical alpine meadow vegetation, and the species composition can be categorized into four taxa according to functional attributes: grasses, sedge, legumes, and miscellaneous grasses [36]. In addition, the vegetation landscape of Hongyuan County, where the study was conducted, was dominated by grasslands, with the average height of the vegetation being about 30 cm, and the natural grasslands were widely distributed, occupying 91.8% of the total area of the county, in which *Kobresia setchwanensis* Hand Mazz, *Elymus nutans* Griseb, *Carex atrofusca* Schkuhr subsp., and *Poa pratensis* L. subsp. constitute the dominant local plant populations. The average bulk density of the soil was 0.2 g/cm^3^.

To ensure the comparability and reliability of the study results, we endeavored to control for differences in environmental variables when selecting the sample plots. The nine selected sample plots were located in the same geographical area on the eastern Tibetan Plateau, with minimal differences in climatic conditions, soil matrices, vegetation types, and geologic environments. The elevation range of these sample plots was between 3282.53 m and 3507 m, with a maximum elevation difference of only 224.47 m. This small elevation difference ensured the consistency of climatic conditions among the sample plots, as the effect of elevation on climate was negligible within the scope of this study. In addition, the soil parent material of all the sample sites is sediment formed in the same geological period, and the geological environment is relatively stable and has not been affected by obvious geological tectonic activities. These conditions provide a more consistent context for studying plant–root–microbe interactions in plateau wetlands and reduce interference due to environmental heterogeneity.

### 4.2. Sample Collection and Analysis

#### 4.2.1. Sample Collection Method

Historical remote sensing image analysis and field research were performed. Normal wetlands with water from May to October each year (nonfreezing period) were defined as water-rich wetlands (W1); wetlands with intermittent water were defined as water-scarce wetlands (W2); and wetlands that once had water but are now water-scarce and arid were defined as aridized wetlands (W3). In selecting the sample plots, we also considered that the climatic conditions, soil matrices, vegetation types, and geological environments among the sample plots did not differ significantly, and the maximum elevation difference was only 224.47 m. These conditions provided a more consistent context for studying plant–root–microbe interactions in plateau wetlands and reduced the interference caused by environmental heterogeneity. After the initial selection of samples, further validation was carried out via soil moisture content measurements, and three sample plots for each moisture condition (nine in total) were selected. Three sample plots of 20 m × 20 m were established in each sample plot, and three sampling points were randomly selected in each sample plot for soil sampling. The use of deep water should be avoided when sampling; if shallow water is encountered, drainage and sampling should be performed. During sampling, after visible plant and animal debris and apomictic material were removed, a clean shovel was used to collect ectorhizosphere soil from 10 cm × 10 cm clods, the soil near the plant roots was gently swept to a plastic bag, and the soil of each sample plot was thoroughly mixed to obtain a composite soil sample, and fine roots and larger plant fragments were removed from the composite soil sample. The soil samples were mixed well and divided into two portions, one of which naturally air dried and preserved for the determination of soil nutrient content, and the other soil sample was quickly preserved in the laboratory at −80 °C for microbial sequencing [81].

#### 4.2.2. Determination of Soil Physical and Chemical Properties

Soil physicochemical properties were determined by referring to the soil agrochemical analysis method of Bao Shidan [82]. Soil total potassium (TK) content was then determined by atomic absorption spectrophotometry. Soil pH was measured using a pH monitor (ThermoOrion-868, ThermoOrion, San, Jose, CA, USA) [7,83]. Soil organic matter content (SOM) was determined by potassium dichromate (K_2_Cr_2_O_7_) oxidation method [84]. Determination of total soil phosphorus (TP) was conducted by wet digestion, treated with H_2_SO_4_ and H_2_O_2_, and then by colorimetric method on a UV2800A UV spectrophotometer (UNIC Inc., Shanghai, China) [85]. Soil total nitrogen (TN) content was determined using an elemental analyzer (FLASH SMART CHNS/0, Bremen, Germany) [86].

#### 4.2.3. Determination of Root Traits in Herbaceous Plants

The collected clods were washed repeatedly; roots were screened; root length (RL, cm), root surface area (RSA, cm^2^), root volume (RV, cm^3^), and root diameter (RD, mm) were determined using an Epson Twain Pro (32 bit) root scanner (Epson Inc, Matsumoto, Japan) and a Win-RHIZO root analysis system (version regular); The roots were then placed in an oven to dry at 80 °C, for 36 h, after which they were removed and weighed to obtain root biomass (RM, mg). Subsequently, root length density (RLD, cm/cm^2^), root surface area density (RSAD, cm^2^/cm^3^), root volume density (RVD, cm^3^/cm^3^), and root biomass density (RMD, mg/cm^3^) were calculated using the following formulas [87]:

(1) RLD = RL/V

(2) RSAD = RSA/V

(3) RVD = RV/V

(4) RMD = RM/V

where V (cm^3^) is the volume of soil from which the roots were extracted.

#### 4.2.4. Measurement of Herbaceous Plant Diversity and Microbial Diversity

For plant diversity indices, four plant diversity indices—Simpson diversity index (D), Shannon‒Wiener diversity index (H), Pielou evenness index (E), and Species richness index (R)—were selected, where R is the number of species observed in the sample, and the rest of the diversity counting formula is as follows [88]:

Simpson diversity index (D):

(5) $$D=1-\sum_{i=1}^{6}{Pi}^{2}$$

Shannon‒Wiener diversity index (H):

(6) $$H=1-\sum_{i=1}^{6}\mathrm{Pi}\ln\mathrm{Pi}$$

Pielou evenness index (E):

(7) $$E\;=\;\frac{H}{\mathrm{lnS}}$$

where Pi is the ratio of the number of individuals of species i to the total number of individuals in the sample site, and S is the total number of individuals of all species in the community.

Microbial diversity index: four microbial diversity indicators—ace index, chao index, Simpson diversity index (D), and Shannon‒Wiener diversity index (H)—were selected and calculated by the cloud tool α-diversity index in the Meiji cloud platform.

#### 4.2.5. Macro-Genome Sequencing Methods for Analyzing Soil Microbial Communities

In this study, we analyzed the diversity and function of soil microbial communities in wetlands of the western Sichuan plateau using macro-genome sequencing technology. The study area was located on the eastern edge of the Tibetan Plateau, and samples were collected from three wetland sample plots with different moisture status, and three soil samples were randomly collected from each sample plot for DNA extraction and physicochemical property analysis. DNA extraction was performed using the E.Z.N.A.^®^ Soil DNA Kit (OMEGA, Norcross, GA, USA), and DNA concentration and purity were determined by NanoDrop2000 (In-T BioTech Inc, Shanghai China). After DNA fragmentation, PE libraries were constructed using the NEXTFLEX Rapid DNA-Seq kit (ThermoFisher, Waltham, MA, USA) and subjected to high-throughput sequencing on the Illumina NovaSeq™ X Plus platform (Yeasen, Shanghai China) [89]. Sequencing data were quality controlled to remove low-quality reads and host contamination and then spliced and assembled using MEGAHIT (version 1.2.9) to screen contigs ≥ 300 bp as the final assembly results. ORFs were predicted by Prodigal (version 2.60), and non-redundant gene sets were constructed using CD-HIT (version 2.60). Use DIAMOND (version 0.8.20) to compare the gene set with NR, KEGG, eggNOG, ARDB, CAZy, SEED and other databases to obtain species annotation information and functional annotation information of genes.

### 4.3. Data Analysis and Graphing

In this study, a variety of statistical and visualization tools were used to provide a comprehensive analysis of plant root traits and the microbial structures and diversities of herbaceous plants under different moisture statuses. One-way analysis of variance (ANOVA) was performed via SPSS 26.0 software on the structure and diversity of the plant root traits and microorganisms of herbaceous plants to assess the significant effect of moisture status. Prism 10.1.2 was used to plot bar graphs of the root trait differences in herbaceous plants to visualize changes in plant root traits under different water conditions. Principal coordinate analysis (PCoA) based on the Bray‒Curtis distance algorithm was used to test the similarity of archaeal, bacterial, and fungal community structure among samples, and the PERMANOVA nonparametric test was used to analyze whether the differences in microbial community structures among sample groups were significant. Key archaeal, bacterial, and fungal taxa at the phylum level were identified between different groups via STAMP analysis. Genes and metabolic pathways of soil archaea, bacteria, and fungi that were significantly associated with plant root traits in herbaceous plants under different moisture statuses were screened via R software (version 4.1.0), and specific functions of these genes in metabolic pathways were identified via the official KEGG website. The results of the descending trend correspondence analysis (DCA) revealed that the gradient lengths of the first sorting axes of the environmental factors, herbaceous plant root traits, and microbial communities were 0.4 and 0.5, respectively, which were less than 3.0. Therefore, redundancy analysis (RDA) was chosen to explore the effects of soil and biological factors on herbaceous plant root traits, as well as on the structures of the soil archaeal, bacterial, and fungal communities, which were visualized via R software. In addition, heatmaps based on the Pearson correlation coefficients of the relationships between key soil microbial phyla and herbaceous plant root traits were produced through an online website https://www.chiplot.online/ (accessed on 25 March 2025) to visualize the correlations between them. Finally, structural equation modeling was performed via SmartPLS4 to reveal potential causal relationships between variables. Other graphics in the article were generated via Origin2024 software.

## 5. Conclusions

This study revealed root–microbe interactions in plateau wetlands under drought conditions. Water-rich wetlands favor root biomass, surface area, and volume, whereas aridized wetlands inhibit these traits and reduce archaeal and bacterial diversity. Root traits correlate with soil pH, total phosphorus, soil organic matter, and microbial diversity. Functional genes such as CS,gltA, G6PD,zwf are associated with root traits linked to drought resilience. Moisture status alters soil organic matter, total nitrogen, total phosphorus, and pH, affecting root characteristics and microbial function. Changes in root traits influence microbial communities, potentially promoting soil nutrient cycling and plant growth. Future work should focus on the functional validation of these microbial genes to elucidate their specific roles in plant drought resilience and nutrient cycling.

## 6. Limitations of This Study

The sample selection for this study was limited to nine wetland sample sites, which, although representative of the study area, may not fully capture the variability across the region. Future studies would benefit from a broader sampling strategy incorporating more sample sites to enhance the robustness and generalizability of the results.

## Figures and Tables

**Table 1:** Soil microbial diversity. W1: water-rich wetlands, W2: water-scarce wetlands, W3: aridized wetlands; values represent diversity indices of soil archaeal, bacterial, and fungal communities under different moisture regimes, which are used to assess the abundance and homogeneity of microbial communities.

Microbial Group	Treatment	Chao1	Shannon‒Wiener Index	Simpson Index
archaea	W1	32	2.32	0.16
	W2	32	2.32	0.17
	W3	32	2.24	0.20
bacteria	W1	159	4.21	0.20
	W2	160	4.23	0.20
	W3	160	4.19	0.20
Fungi	W1	8	2.02	0.14
	W2	8	2.04	0.13
	W3	8	2.00	0.14

## Data Availability

The data supporting the study findings are available on request from the corresponding author.

## Supplementary Materials

The following supporting information can be downloaded at https://www.mdpi.com/article/10.3390/plants14152413/s1: Table S1. Metabolic pathways involved in key functional genes.

## References

[1] Chen Y., Lyu X. (2003). Research Directions in Wetland Function and Wetland Science. Wetl. Sci..

[2] Sandi S.G., Rodriguez J.F., Saintilan N., Wen L., Kuczera G., Riccardi G., Saco P.M. (2020). Resilience to drought of dryland wetlands threatened by climate change. Sci. Rep..

[3] Liu C., Siri M., Li H., Ren C., Huang J., Feng C., Liu K. (2023). Drought is threatening plant growth and soil nutrients of grassland ecosystems: A meta-analysis. Ecol. Evol..

[4] Zhao Z., Zhang Y., Liu L., Liu F., Zhang H. (2015). Recent changes in wetlands on the Tibetan Plateau: A review. J. Geogr. Sci..

[5] Kang D., Chen Y., Feng S., Liu Q., Zou S. (2025). Microbial community diversity and assembly processes in the aridification of wetlands on the Qinghai-Tibet Plateau. iScience.

[6] Ouyang L., Ba S., La D., Liu X., Liu G., Liu W., Ding B. (2023). Analysis of Plant and Soil Bacterial Diversity in Wetlands on the Tibetan Plateau and Their Influencing Factors. Acta Bot. Sin..

[7] Jiang W., Xiong M., Zou S., Kang D. (2024). Microbial genes for degrading plant-derived carbon are a key factor affecting soil respiration and temperature sensitivity in plateau peatlands. Pedosphere.

[8] Wang T., Zhang Y., Zhao Z. (2020). Vegetation community structure and soil nutrient changes in degraded alpine wetlands on the Qinghai-Tibet Plateau. J. Grassl. Sci..

[9] Xiong M., Jiang W., Zou S., Kang D., Yan X. (2023). Microbial carbohydrate-active enzymes influence soil carbon by regulating the of plant- and fungal-derived biomass decomposition in plateau peat wetlands under differing water conditions. Front. Microbiol..

[10] Wu J., Zeng R., Yin H., He Y., Tang Z., Song S. (2024). Mechanisms of plant responses to water stress. Mod. Agric. Res..

[11] He P.-C., Qing Y.E. (2019). Plant Functional Traits: From Individual Plant to Global Scale. J. Trop. Subtrop. Bot..

[12] Liu L., Huang X., Zhang J., Cai Z., Jiang K., Chang Y. (2020). Deciphering the relative importance of soil and plant traits on the development of rhizosphere microbial communities. Soil Biol. Biochem..

[13] Wardle D.A., Bardgett R.D., Klironomos J.N., Setälä H., van der Putten W.H., Wall D.H. (2004). Ecological Linkages Between Aboveground and Belowground Biota. Science.

[14] Delgado-Baquerizo M., Fry E.L., Eldridge D.J., de Vries F.T., Manning P., Hamonts K., Kattge J., Boenisch G., Singh B.K., Bardgett R.D. (2018). Plant attributes explain the distribution of soil microbial communities in two contrasting regions of the globe. New Phytol..

[15] Chen D., Pan Q., Bai Y., Hu S., Huang J., Wang Q., Naeem S., Elser J.J., Wu J., Han X. (2016). Effects of plant functional group loss on soil biota and net ecosystem exchange: A plant removal experiment in the Mongolian grassland. J. Ecol..

[16] Bever J.D. (2003). Soil community feedback and the coexistence of competitors: Conceptual frameworks and empirical tests. New Phytol..

[17] van der Putten W.H., Bardgett R.D., Bever J.D., Bezemer T.M., Casper B.B., Fukami T., Kardol P., Klironomos J.N., Kulmatiski A., Schweitzer J.A. (2013). Plant–soil feedbacks: The past, the present and future challenges. J. Ecol..

[18] Van Der Heijden M.G., Bakker R., Verwaal J., Scheublin T.R., Rutten M., Van Logtestijn R., Staehelin C. (2006). Symbiotic bacteria as a determinant of plant community structure and plant productivity in dune grassland. FEMS Microbiol. Ecol..

[19] van der Putten W.H., Bradford M.A., Pernilla Brinkman E., van de Voorde T.F., Veen G.F. (2016). Where, when and how plant–soil feedback matters in a changing world. Funct. Ecol..

[20] Van Der Heijden M.G., Bardgett R.D., Van Straalen N.M. (2008). The unseen majority: Soil microbes as drivers of plant diversity and productivity in terrestrial ecosystems. Ecol. Lett..

[21] Bauer J.T., Mack K.M.L., Bever J.D. (2015). Plant-soil feedbacks as drivers of succession: Evidence from remnant and restored tallgrass prairies. Ecosphere.

[22] Bever J.D., Westover K.M., Antonovics J. (1997). Incorporating the Soil Community into Plant Population Dynamics: The Utility of the Feedback Approach. J. Ecol..

[23] McNear D. (2013). The rhizosphere-roots, soil and everything in between. Nat. Educ. Knowl..

[24] Liang Q., Liao H., Yan X. (2007). Quantitative analysis of plant root morphology. Acta Bot. Sin..

[25] Guo Y., Yao J., Dong Y., Yan J., Yang N., Feng Y., Wei X., Liang W. (2023). Root distribution characteristics of pure stands and mixed forests of *Pinus tabulaeformis* and *Robinia pseudoacacia*. J. Appl. Ecol..

[26] Wang X. (2015). Physiological Responses of Four Types of Grasses to Drought Stress. Master’s Thesis.

[27] Cheng J., Zhang Y. (2012). Biomass allocation strategies of two herbaceous plants in arid regions under water stress. Arid. Zone Res..

[28] Liu J., Li X., Yao M. (2021). Research Progress on the Construction of Plant Rhizosphere Microbial Communities. Acta Microbiol. Sin..

[29] Wang C., Kuzyakov Y. (2024). Mechanisms and implications of bacterial–fungal competition for soil resources. ISME J..

[30] Zhang F., Hou Y., Ao Y., Shen J., Jin K. (2021). Root-soil interactions under soil compaction stress. J. Nutr. Fertil. Sci..

[31] Xi N., De Long J.R., Davison J., Kardol P., Forero L.E., Zobel M., Semchenko M. (2025). Plant–soil microbial interactions as modulators of species coexistence and productivity. Trends Ecol. Evol..

[32] Zhang X., Wang L., Liu H., Liu D., Wang W., Liang C., Recknagel F. (2014). Nutrient absorption kinetics of three emergent aquatic plants: *Phragmites australis*, *Typha angustifolia*, and *Juncus effusus*. Acta Ecol. Sin..

[33] Wu S., Wang G., Yang L., Liu S. (2024). Research Progress on the Impact of Drought on the Structure and Function of Forest Ecosystems. World For. Res..

[34] Li F., Pei N., Shi Z., Luo S., Tang Y., Liu X., Chen Z., Sun B. (2019). Secondary Forest and Artificial Forest Root Biomass, Morphological Characteristics, Nutrients, and Their Relationship with Soil Nutrients. J. Ecol. Environ..

[35] Zhe H., Luo Y., Zhang H., Sun F. (2011). Study on the Relationship between Root Biomass of Saplings of *Alnus mandshurica*, *Quercus mongolica*, and *Ulmus parvifolia* in Daqing Gou and Soil Nutrients and pH Value. Arid. Zone Resour. Environ..

[36] Wang C., Long R., Ding L. (2004). The influence of functional group diversity and composition of different grassland types on plant community productivity in alpine meadows. Biodivers. Sci..

[37] Liu Y., Li F., Sun Q., Xie Y. (2013). Advances in the study of soil microorganisms in wetland ecosystems. J. Appl. Environ. Biol..

[38] Feng S., Zhang H., Wang Y., Bai Z., Zhuang G. (2009). Analysis of fungal community structure in the soil of Zoige Alpine Wetland. Acta Ecol. Sin..

[39] Tian J., Zhu Y., Kang X., Dong X., Li W., Chen H., Wang Y. (2012). Effects of drought on the archaeal community in soil of the Zoige wetlands of the Qinghai–Tibetan plateau. Eur. J. Soil Biol..

[40] Wang Y., Ren L., Weng B., Ying C., Huang Y. (2005). Ecological effects of soil drought stress on soil microorganisms in round-leaved cassia. J. Xiamen Univ. (Nat. Sci. Ed.).

[41] Baker B.J., De Anda V., Seitz K.W., Dombrowski N., Santoro A.E., Lloyd K.G. (2020). Diversity, ecology and evolution of Archaea. Nat. Microbiol..

[42] Gupta A., Rico-Medina A., Caño-Delgado A.I. (2020). The physiology of plant responses to drought. Science.

[43] Zhang H., Liu H., Zhao J., Li G., Lai X., Li J., Wang H., Yang D. (2018). Response of fungal community structure in Baikal needle grassland soil to nitrogen and water addition. Acta Ecol. Sin..

[44] Wang Z., Wang W., Ma Z., Chen K., Ben H., Min K., Qiao Y. (2023). Effects of different grass species growth on soil nutrients and microbial communities in the Gansu *Artemisia annua* community. J. Grassl. Sci..

[45] Zhang G., Bai J., Jia J., Wang W., Wang D., Zhao Q., Wang C., Chen G. (2023). Soil microbial communities regulate the threshold effect of salinity stress on SOM decomposition in coastal salt marshes. Fundam. Res..

[46] Jiao J., Fu X., Zhang S., Liu W., Zhou J., Wu X., Lin X., Tian Y., Tang G., Li P. (2023). Analysis of the physicochemical properties and microbial community structure of rhizosphere soil in Sichuan pepper trees of different ages. J. Northwest For. Univ..

[47] Yan F., Zhao X., Shao L., Wang X., Liang Y., Chen Y. (2025). Effects of Different Vegetation Restoration Types on Soil Microbial Community Structure in the Restoration Area of the Jibei Quarry. Environ. Sci..

[48] Tong Y., Zhang C., Yu Y., Cao Q., Yang Z., Zhang X., Wang M., Dong Q. (2024). Response of soil microbial characteristics in perennial high-altitude cultivated grasslands to short-term nitrogen addition. Environ. Sci..

[49] Herms C.H., Hennessy R.C., Bak F., Dresbøll D.B., Nicolaisen M.H. (2022). Back to our roots: Exploring the role of root morphology as a mediator of beneficial plant-microbe interactions. Environ. Microbiol..

[50] Jousset A., Schulz W., Scheu S., Eisenhauer N. (2011). Intraspecific genotypic richness and relatedness predict the invasibility of microbial communities. ISME J..

[51] van Elsas J.D., Chiurazzi M., Mallon C.A., Elhottovā D., Krištůfek V., Salles J.F. (2012). Microbial diversity determines the invasion of soil by a bacterial pathogen. Proc. Natl. Acad. Sci. USA.

[52] Yuan R., Liu L., Zhang R., Fan S. (2020). Advances in Research on the Mechanisms of Interactions Between Plant Rhizosphere Exudates and Soil Microorganisms. Chin. Agric. Sci. Bull..

[53] Tomazelli D., Peron R.A.d.S., Mendes S.D.C., Pinto C.E., Baldissera T.C., Baretta D., Mendes L.W., Goss-Souza D., Klauberg-Filho O. (2024). Plant diversity and root traits shape rhizosphere microbial communities in natural grasslands and cultivated pastures. Rhizosphere.

[54] Li Y.-X., Rao Y.-Z., Qi Y.-L., Qu Y.-N., Chen Y.-T., Jiao J.-Y., Shu W.-S., Jiang H., Hedlund B.P., Hua Z.-S. (2021). Deciphering Symbiotic Interactions of “*Candidatus* Aenigmarchaeota” with Inferred Horizontal Gene Transfers and Co-occurrence Networks. mSystems.

[55] Cui X., Lin X., Li J., Zhang H., Han Y. (2023). Diversity, functional characteristics, and applications of stress-tolerant actinomycetes in environmental remediation. Acta Microbiol. Sin..

[56] Wang S., Chang S., Li X., Zhang Y. (2021). Soil fungal diversity and community structure in the Tianshan Forest Region. Acta Ecol. Sin..

[57] Wu L.K., Lin X.M., Lin W.X. (2014). Research progress and prospects on plant-soil-microbial interactions mediated by root exudates. Acta Bot. Sin..

[58] Delgado-Baquerizo M., Maestre F.T., Reich P.B., Jeffries T.C., Gaitan J.J., Encinar D., Berdugo M., Campbell C.D., Singh B.K. (2016). Microbial diversity drives multifunctionality in terrestrial ecosystems. Nat. Commun..

[59] Zhu Y.-G., Zhao Y., Zhu D., Gillings M., Penuelas J., Ok Y.S., Capon A., Banwart S. (2019). Soil biota, antimicrobial resistance and planetary health. Environ. Int..

[60] Wagg C., Schlaeppi K., Banerjee S., Kuramae E.E., Van Der Heijden M.G.A. (2019). Fungal-bacterial diversity and microbiome complexity predict ecosystem functioning. Nat. Commun..

[61] Song Z.H., Tian P., Pei T. (2024). Overview of the role of soil microorganisms in soil improvement and remediation. Grassl. Sci..

[62] Kang D., Li Y., Ma L., Zou S. (2023). Landslide scales affect soil organic carbon accumulation by influencing microbial decomposition of plant-derived carbon after earthquakes. Ecol. Indic..

[63] Pantigoso H.A., Ossowicki A., Stringlis I.A., Carrión V.J. (2025). Hub metabolites at the root–microbiome interface: Unlocking plant drought resilience. Trends Plant Sci..

[64] Shah B.A., Kasarlawar S.T., Phale P.S. (2022). Glucose-6-Phosphate Dehydrogenase, ZwfA, a Dual Cofactor-Specific Isozyme Is Predominantly Involved in the Glucose Metabolism of *Pseudomonas bharatica* CSV86T. Microbiol. Spectr..

[65] Abedini D., Jaupitre S., Bouwmeester H., Dong L. (2021). Metabolic interactions in beneficial microbe recruitment by plants. Curr. Opin. Biotechnol..

[66] de Vries F.T., Griffiths R.I., Knight C.G., Nicolitch O., Williams A. (2020). Harnessing rhizosphere microbiomes for drought-resilient crop production. Science.

[67] Suarez-Fernandez M., Ferreira J.J., Campa A. (2025). Impact of Farming System on Soil Microbial Communities Associated with Common Bean in a Region of Northern Spain. Plants.

[68] Aleksieienko I., Fernandes Hertel M., Reilhan J., De Castro M., Légeret B., Caixeta Oliveira H., Reiter I.M., Santaella C. (2025). Soil-Gradient-Derived Bacterial Synthetic Communities Enhance Drought Tolerance in *Quercus pubescens* and *Sorbus domestica* Seedlings. Plants.

[69] Liu Q., Li W., Jia Z., An W., Zhao G., Su Q., Li Y. (2023). Research Progress and Hot Topics Analysis on the Adaptive Mechanisms of Plant Root Systems Under Drought Stress. Jiangsu Agric. Sci..

[70] Ren H., Lu H. (2025). The issue of appropriate trees for appropriate locations in ecosystem restoration. J. Trop. Subtrop. Bot..

[71] Maurel C., Nacry P. (2020). Root architecture and hydraulics converge for acclimation to changing water availability. Nat. Plants.

[72] Yang M., Guo H., Duan G., Wang Z., Fan G., Li J. (2024). Research Progress on the Role and Mechanism of Arbuscular Mycorrhizal Fungi in Enhancing Plant Stress Resistance and Soil Improvement. Chin. Powder Technol..

[73] Liang S., Li H., Wu H., Yan B., Song A. (2023). Microorganisms in coastal wetland sediments: A review on microbial community structure, functional gene, and environmental potential. Front. Microbiol..

[74] Ma J., Gao C., Yi X., Wu Y., Li J., Zeng X., Cai J. (2023). Analysis of soil microbial characteristics in different habitats during the ecological restoration process of Haifeng Wetland, Guangdong Province. Environ. Sci..

[75] Ma H., Zhao Y., Yang K., Wang Y., Zhang C., Ji M. (2022). Application oriented bioaugmentation processes: Mechanism, performance improvement and scale-up. Bioresour. Technol..

[76] Ruan Z., Chen K., Cao W., Meng L., Yang B., Xu M., Xing Y., Li P., Freilich S., Chen C. (2024). Engineering natural microbiomes toward enhanced bioremediation by microbiome modeling. Nat. Commun..

[77] Jing J., Garbeva P., Raaijmakers J.M., Medema M.H. (2024). Strategies for tailoring functional microbial synthetic communities. ISME J..

[78] Jing J., Cong W.-F., Bezemer T.M. (2022). Legacies at work: Plant–soil–microbiome interactions underpinning agricultural sustainability. Trends Plant Sci..

[79] Hartmann M., Six J. (2023). Soil structure and microbiome functions in agroecosystems. Nat. Rev. Earth Environ..

[80] Yang D., Hu L., Song X., Wang C. (2021). Effects of rainfall variability on litter mass and decomposition of different plant functional groups in alpine meadows. Acta Bot. Sin..

[81] Chen Y., Xie Z. (2018). Effects of storage conditions on soil biochemical indicators and the application of stored soil samples. Acta Pedol. Sin..

[82] Bao S. (2000). Soil Agrochemical Analysis.

[83] Yan G., Xing Y., Han S., Zhang J., Wang Q., Mu C. (2020). Long-time precipitation reduction and nitrogen deposition increase alter soil nitrogen dynamic by influencing soil bacterial communities and functional groups. Pedosphere.

[84] Komy Z.R. (1995). Comparative Study of Titrimetric and Gravimetric Methods for the Determination of Organic Carbon in Soils. Int. J. Environ. Anal. Chem..

[85] Fu B., Liu S., Chen L., Lü Y., Qiu J. (2004). Soil quality regime in relation to land cover and slope position across a highly modified slope landscape. Ecol. Res..

[86] Xiong W., Zhao Q., Zhao J., Xun W., Li R., Zhang R., Wu H., Shen Q. (2015). Different Continuous Cropping Spans Significantly Affect Microbial Community Membership and Structure in a Vanilla-Grown Soil as Revealed by Deep Pyrosequencing. Microb. Ecol..

[87] Wang P. (2021). The Influence of Root Systems of Typical Herbaceous Plants in the Loess Plateau on Soil Infiltration and Spatial Differences. Master’s Thesis.

[88] Zeng Y., Mai Reziya ·W., Abdulheli ·Y., Aikebai ·N.M., Liang Y., Liu Y. (2025). Plant Diversity and Community Stability in Different Habitats Along the Upper Reaches of the Tarim River, Xinjiang. J. Ecol..

[89] Modi A., Vai S., Caramelli D., Lari M., Mengoni A., Bacci G., Fondi M. (2021). The Illumina Sequencing Protocol and the NovaSeq 6000 System. Bacterial Pangenomics: Methods and Protocols.

