# Supplementary material for: Drought Modulates Root–Microbe Interactions and Functional Gene Expression in Plateau Wetland Herbaceous Plants
Source: Plants (Basel). 2025 Aug 4;14(15):2413. doi: 10.3390/plants14152413 (PMC12349268; doi:10.3390/plants14152413)
Supplement: Supplementary file 1 [file plants-14-02413-s001.zip › plants-3756313-supplementary.pdf]

Supplementary Material

Table S1. Metabolic pathways involved in key functional genes

This table lists microbial key functional genes and the metabolic pathways they are involved in that are relevant to plant root function and drought tolerance. Each gene or combination of genes plays an important role in a specific metabolic pathway, and their functional descriptions detail the specific roles of these genes in plant-microbe interactions.

gene name	Metabolic pathways	Description of thefunction
fabG,OAR1<br>ACAT,atoB<br>asnB,ASNS<br>CS,gltA<br>G6PD,zwf<br>leuA,IMS<br>glnA,GLUL	Biosynthesis of secondary metabolites	fabG,OAR1 is involved in the synthesis of 3-oxoacyl-[acyl-carrier protein] reductase; ACAT,atoB generates acetyl-CoA C-acetyltransferase; asnB,ASNS synthesizes asparagine synthase; CS. gltA synthesizes citrate synthase to drive the tricarboxylic acid cycle; G6PD,zwf synthesizes glucose-6-phosphate 1-dehydrogenase, leuA,IMS synthesizes 2-isopropylmalate synthase; glnA,GLUL synthesizes glutamine synthetase together support the synthesis of secondary metabolites and metabolic homeostasis.
ACAT,atoB<br>CS,gltA<br>mcmA1<br>G6PD,zwf	Carbon metabolism	ACAT,atoB catalyzes the conversion of carbon sources to acetyl-CoA C-acetyltransferase; CS, gltA synthesizes citrate synthase to drive the tricarboxylic acid cycle; mcmA1 synthesizes methylmalonyl-CoA mutase; G6PD,zwf synthesizes glucose-6- phosphate 1-dehydrogenase; G6PD,zwf synthesize glucose-6-phosphate 1-dehydrogenase, which work together to maintain carbon metabolic balance and provide raw materials for biosynthesis.
asnB,ASNS<br>CS,gltA<br>mcmA1<br>glnA,GLUL<br>leuA,IMS	Biosynthesis of amino acids	asnB,ASNS synthesize asparagine synthase; CS,gltA synthesize citrate synthase to drive the tricarboxylic acid cycle; mcmA1 synthesize methylmalonyl-CoA m

		utase; glnA,GLUL synthesize glu tamine synthetase to regulate nit rogen balance; leuA,IMS synthes izes 2-isopropylmalate synthase, which together maintain amino a cid metabolic homeostasis.
ATPF1A,atpA<br>ATPF1B,atpD<br>nuoD<br>nuoF<br>nuoH<br>nuoL<br>nuoN	Oxidative phosphorylation	ATPF1A,atpA is involved in the synthesis of the F-type H+/Na+-transporting ATPase subunit alpha; ATPF1B,atpD synthesizes the F-type H+/Na+-transporting ATPase subunit beta, which directly catalyzes ATP synthesis; and the nuoD/ F/H/L/N and other genes encode NADH dehydrogenase subunits that mediate electron transport and proton pumping.
ACAT,atoB<br>ACSL,fadD<br>fabF,OXSM,CEM1<br>fabG,OAR1	Fatty acid metabolism	ACAT,atoB provide acetyl-C oA C-acetyltransferase as raw m aterial for fatty acid synthesis; ACSL,fad synthesize Dlong-chai n-fatty-acid--CoA ligase to prom ote the subsequent metabolism; f abF,OXSM,CEM1 synthesize 3-o xoacyl-[acyl-carrier-protein] synt hase II regulates fatty acid chai n extension and structural modif ication; fabG,OAR1 is involved in the synthesis of 3-oxoacyl-[ac yl-carrier protein] reductase; thes e genes synergistically regulate t he whole process of fatty acid metabolism.
CS,gltA	Citrate cycle (TCA cycle)	CS,gltA encodes citrate synt hase to drive the tricarboxylic a cid cycle; the synthase catalyzes the TCA cycle initiation reactio n (acetyl coenzyme A + oxaloac etate → citrate), and its encode d aconitase drives the citrate/iso citrate conversion, which synergi stically guarantees the efficient operation of the TCA cycle, wit h a continuous supply of energy

		(ATP/NADH) and carbon-skeleton precursors.
G6PD,zwf	Pentose phosphate pathway	G6PD,zwf synthesizes glucose-6-phosphate 1-dehydrogenase to provide cells with reducing power and nucleic acid precursors.
glnA,GLUL	Nitrogen metabolism	glnA,GLUL catalyze the synthesis and catabolism of glutamine, respectively, and synergistically realize the organic conversion of inorganic nitrogen and nitrogen recycling and reuse, and jointly maintain the balance of cellular nitrogen metabolism and amino acid supply.
